# Early Upper Paleolithic colonization across Europe: Time and mode of the Gravettian diffusion

**DOI:** 10.1371/journal.pone.0178506

**Published:** 2017-05-24

**Authors:** Nuno Bicho, João Cascalheira, Célia Gonçalves

**Affiliations:** ICArEHB (Interdisciplinary Center for Archaeology and the Evolution of Human Behavior), Faculdade de Ciências Humanas e Sociais, Universidade do Algarve, Campus de Gambelas, Faro, Portugal; Max Planck Institute for the Science of Human History, GERMANY

## Abstract

This study presents new models on the origin, speed and mode of the wave-of-advance leading to the definitive occupation of Europe’s outskirts by Anatomically Modern Humans, during the Gravettian, between c. 37 and 30 ka ago. These models provide the estimation for possible demic dispersal routes for AMH at a stable spread rate of c. 0.7 km/year, with the likely origin in Central Europe at the site of Geissenklosterle in Germany and reaching all areas of the European landscape. The results imply that: 1. The arrival of the Gravettian populations into the far eastern European plains and to southern Iberia found regions with very low human occupation or even devoid of hominins; 2. Human demography was likely lower than previous estimates for the Upper Paleolithic; 3. The likely early AMH paths across Europe followed the European central plains and the Mediterranean coast to reach to the ends of the Italian and Iberian peninsulas.

## Introduction

The occupation of Europe by Anatomically Modern Humans (AMH) started sometime before 40 thousand years ago [1, 2], replacing the previous Neanderthal populations [3, 4]. The human occupation of the full ice-free European territory was, however, accomplished only with the Gravettian techno-complex replacing the previous Aurignacian tradition, and in certain marginal regions replacing either Neanderthal populations [5] or populating new uninhabited territories [3, 6].

A quarter of century ago, Otte and Keeley published a paper on Current Anthropology [7] advocating the impact of regional studies on the perception of the expansion of Upper Paleolithic techno-complexes through the European continent. This paper, based on the earliest non-calibrated radiocarbon dates and general location of Upper Paleolithic sites, provided a first insight on the time and direction of AMH expansion in Europe. Those authors concluded that the origin of the Gravettian, in particular, was in central Europe, probably Austria or Germany, some 27 ka radiocarbon years ago, expanding at different rates and arriving at the eastern and eastern European limits at about 20 ka radiocarbon years ago.

Since then, various wave-of-advance models for Pleistocene humans, generally based on Reaction-Dispersal Models [8], were built, dealing with the AMH “Out of Africa” [9, 10], the replacement of Neanderthals by AMH [11], the human replacement in Europe after the Last Glacial Maximum [12] or the Clovis rapid colonization of North America [13].

In this study, we focus on four main aspects related to the spread of early AMH in Europe and focused only on the Gravettian techno-complex because it was the first technological phase present all over the European territory: the contact and replacement of pre-AMH by AMH in the marginal areas or Europe; the time and speed of dispersal of the Gravettian techno-complex; if this advance was predominantly the result of demographic expansion or cultural diffusion; and, finally, the possible routes that AMH with Gravettian technology followed across Europe.

We analyzed the spatial distribution of early Gravettian calibrated AMS dates across Europe, from a total of 33 sites spreading from Russia to Southern Portugal (S1 Table). To measure the advance speed rate (see S1 Table) we followed the statistical procedures outlined by Fort and colleagues in their recent studies of other human prehistoric expansions [8, 14–16]. We did not, however, use neither of the two calculation methods for distance used by those authors: the great circle approach [14], based on the models developed by Fort [12, 16–18] and the variant of the shortest path approach [14]. We used, instead, a new method based on the GIS-based Least-Cost Path assessment that includes topographic and landscape data to estimate the best route between two points.

## Materials and methods

### Radiocarbon data

S1 Table includes all the earliest AMS dates for Gravettian horizons across Europe present in the Leuven Radiocarbon Palaeolithic Europe Database, Version 20 [19]. We did not include conventional radiocarbon results because, as has been frequently published, those results are not nearly as reliable as those from the AMS methods; we also did not use any dates with standard errors larger than 500 years. Still, we are aware that even these have various problems that are related to both the type of sample (i.e., charcoal, bone and shell) and the type of pretreatment that each laboratory performs (e.g.,[20, 21–25]).

We used only the oldest Gravettian single date from each site. We also rejected contexts whose cultural or chronological attributions were equivocal (e.g., a Gravettian horizon dated to 45 ka or a date coming from a layer attributed to “Aurignacian/Gravettian” or to “Gravettian?”). Finally, we limited the chronology to dates older than 27 ka radiocarbon years.

We were able to filter a total of 33 sites dated to between 37.5 and 30 ka cal BP. Calibration of the AMS dates was carried out using the OxCal software online (https://c14.arch.ox.ac.uk) and the IntCal13 calibration curve for the northern hemisphere [26].

To define and confirm the oldest Gravettian site we used the Order command and the Difference function in the OxCal software. The Order command provides information on the probability distribution for the difference between two dates. With these two functions, we were able to compare all sites and define the oldest sites, as well as to check for the probability of chronological overlap between those earlier sites (S2 Table). Thus, we were able to check that there are three potential locations as the oldest Gravettian sites, in order of antiquity: Buran Kaya III, Geissenklosterle and Krems-Hundssteig. The Difference function indicates that there is a probability of a couple of hundred years of overlap between the first two (-2194/271), and of about a millennium between the latter two sites (-2200/1087). There is no overlap between Buran Kaya III and Krems-Hundssteig (-2826/-116). The Order command confirms that Buran Kaya III is the oldest site, followed by Geissenklosterle that has an 80% probability of being older that Krems-Hundssteig.

### GIS methods

All spatial calculations were done using ArcGIS 10.4.1 by ESRI. Cost-distance modeling was accomplished using the elevation dataset from the Shuttle Radar Topography Mission (SRTM) with a resolution of 1 arc-second (i.e., 30m square-grid) available at https://earthexplorer.usgs.gov.

**Fig 1:**
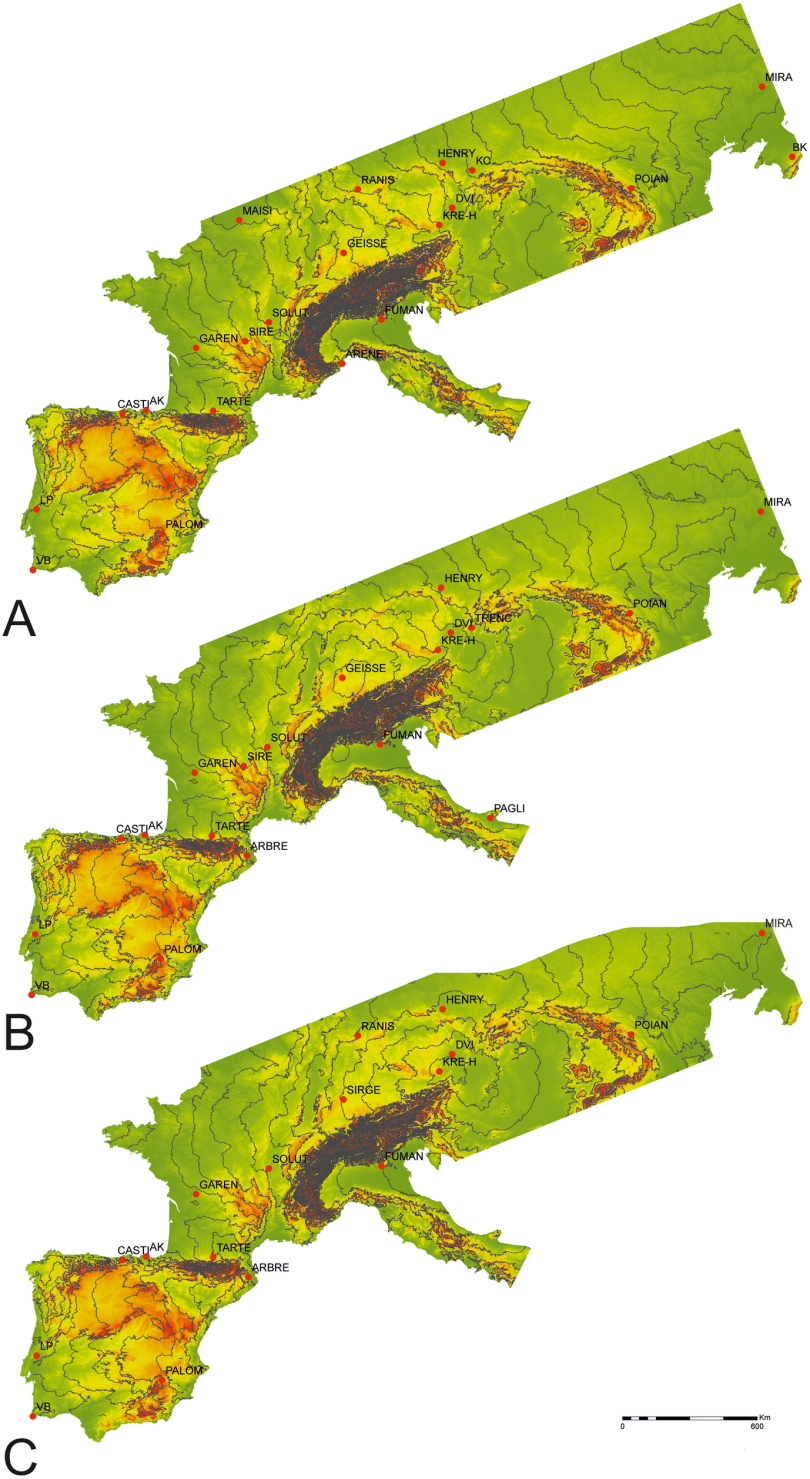
Map with the 150 km isopleths for the Cost-distance models for A) Buran Kaya III; B) Geissenklosterle; C) Krems-Hundssteig.

The software first creates an Accumulated Cost Surface where each cell in the new raster has a value that accumulates the cost of moving outward from the origin until reaching the destination, while storing the backlink raster that represent the path of least cost. The cost surface was acquired using the slope raster, generated with the Slope tool in the Spatial Analyst toolbox using degrees as the output measurement, and a vertical factor table that is used by the Path Distance tool to convert slope in time. This was developed with the Tobler´s hiking function [27] to find the most time-efficient paths between each of the oldest sites and all the remaining sites. Tobler´s function assigns a temporal cost per slope degree and is used in many archaeological Least-Cost Path studies (e.g., [28, 29–31]). Least-Cost Paths presented in Fig 1 were generated from the Accumulated Cost Surfaces and backlink raster by using the Cost Path tool, and Accumulated Cost Surface converted to vector lines using the Raster to Polyline tool. In this type of analysis, the eight neighbors of a raster cell are evaluated and the generated path moves to the cells with the smallest accumulated or cost value. This process is repeated multiple times until the source and destination are connected. The completed path is the smallest sum of raster cell values between the two points and it has the lowest cost.

### Measuring the wave-of-advance

Since we were not able to define a single oldest site, due to the probability of chronological overlap among the three sites, we considered each of those locations individually as the possible epicenter for the origin and expansion of the Gravettian techno-complex. Based on each possible origin, and following the distance of 150 km radius used by Fort, Pujol and Cavalli-Sforza [12] for Paleolithic waves-of-advance, we computed 150 km isopleths starting in Buran Kaya III, Geissenklosterle and Krems-Hundssteig, using a single approach for calculation: the Distance-cost method.

After the distance models were calculated (Fig 1), we plotted site locations and selected, for the estimation of spread velocity, only one site within each two isopleths, the one with the oldest date.

We then calculated the distance between the origin site and all other sites using the Least-Cost Path method (Table 1). These models compute the minimal straight-line distance between two sites, incorporating earth curvature into the calculation. The Least-Cost Path data also took into account the Digital Elevation Model and Slope values (see GIS methods below) to estimate the least-cost route between the three possible origin sites and each one of the remaining sites.

**Table 1:** Least-Cost Path distances from the three earliest sites to the sites included in each regression.

Site	Code	Mean calibrated age (BP)	Least-Cost Path from Buran Kaya (Km)	Least-Cost Path from Geissenklosterle (Km)	Least-Cost Path from Krems-Hundssteig (Km)
Buran Kaya	BK	38528	-		-
Geissenklosterle	GEISSE	37569	3701	-	-
Krems-Hundssteig	KRE-H	37124	3062	614	-
Ranis 4 Ilsenhohle	RANIS	35655	3327	-	506
Dolni Vestonice IIa	DVI	35550	2946	751	130
Fumane	FUMAN	35479	3200	790	1111
Henrykow 15	HENRY	35477	2833	784	437
Trencianske Bohuslavice-Pod Tureckom	TRENC	34058	-	880	-
EL Castillo	CASTI	33887	5613	1994	2530
Le Sire	SIRE	33465	4533	876	-
Maisieres Canal, champ de fouille	MAISI	33261	4122	-	-
Lapa do Picareiro	LP	33230	6543	2927	3459
Komarowa Cave	KC	32526	2705	-	-
Vale Boi	VB	32372	6537	2922	3450
Les Garennes	GAREN	32324	4793	1136	1668
Solutre-J-10	SOLUT	32319	4357	700	1231
Tarte	TARTE	32308	5013	1397	1930
Arbreda	ARBRE	32227	-	1345	1878
Paglicci	PAGLI	32157	-	1472	-
Palomar	PALOM	31983	5744	2129	2662
Antonilako Koba	AK	31348	5457	1839	2374
Mira	MIRA	31315	736	2888	2559
Grotta Arene Candide	ARENE	31263	3554	-	-
Piana Ciresului	POIAN	31236	1577	1774	1169
Sirgenstein	SIRG	31184			617

Finally, we also computed the time interval between the mean calibrated date of each of the three earliest sites, and the mean calibrated date of each one of the remaining sites.

Following the arguments of Fort [15], Hamilton and Buchanan [13], and Jerardino et al. [14] (i.e., there is likely much more error in the measurement of time than in the measurement of distance), to estimate the spread rate of the Gravettian techno-complex, time intervals (y axis) were plotted by distance (x axis), and a linear regression was fitted for each distance calculation model. Since the number of sites is fairly low, each linear regression was computed with an 80% confidence-level interval. Using the slope value from each regression, the speed and error of propagation were calculated following Jerardino et al. (equations 2 through 4).

## Results

Differences among the three distance models are very clear, both in terms of the calculated distances between each site and the origin point but also, and consequently, in the set of sites selected to be included in each regression (Table 1). This is mostly due to the fact that because a Cost-distance estimation is used, sites located beyond an important mountain range such as the Alps or the Pyrenees show an increase of over 60% in distance over the Geodesic distance: a good example is the case of Fumane cave where that distance drastically increases from slightly less than 350 kms to close to 800 kms. In all other cases, however, distances tend to increase less than 50%.

**Fig 2:**
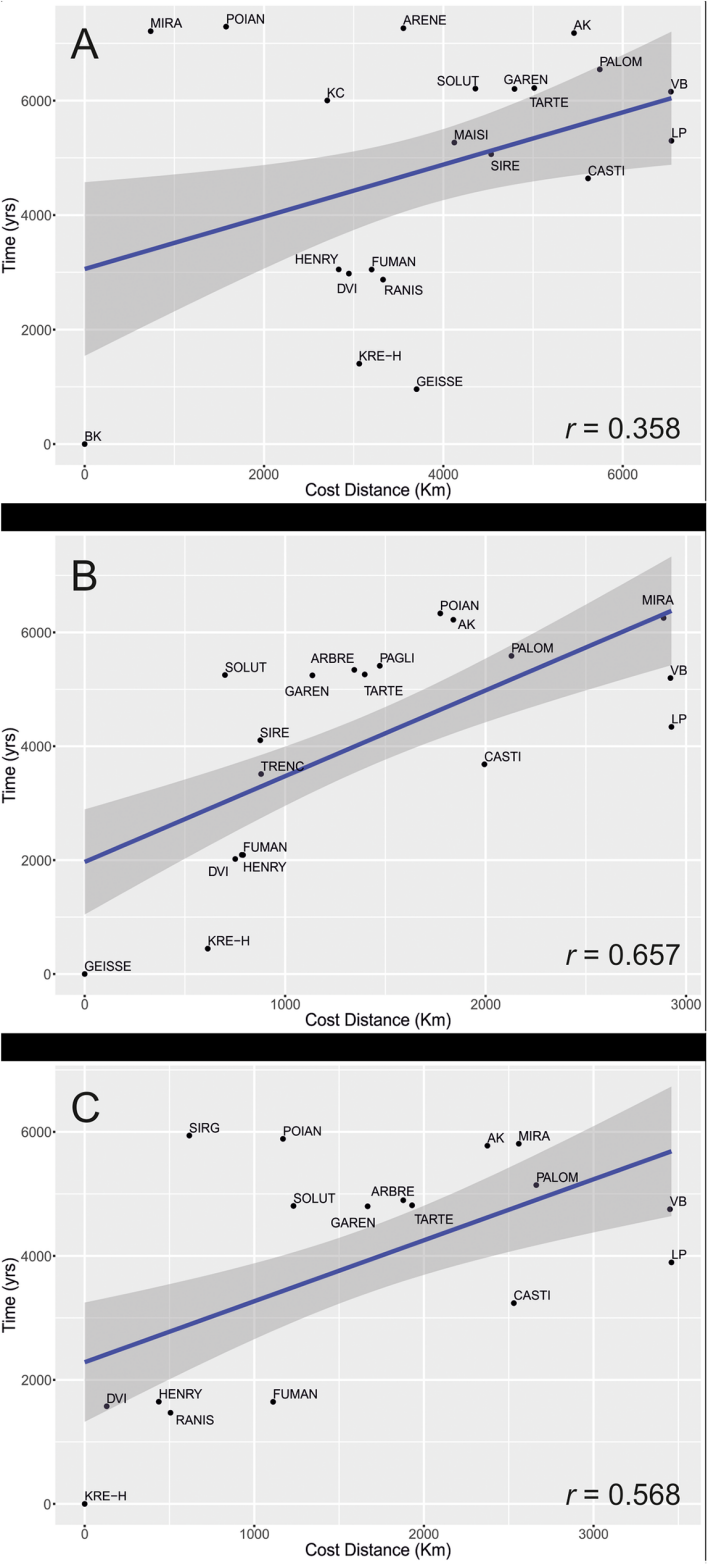
Linear regression fits to determine the speed of advance of the Gravettian for: A. Buran Kaya III model; B. Geissenklosterle model; C. Krems-Hundssteig model. Time, distances and site codes are listed in Table 1.

**Fig 3:**
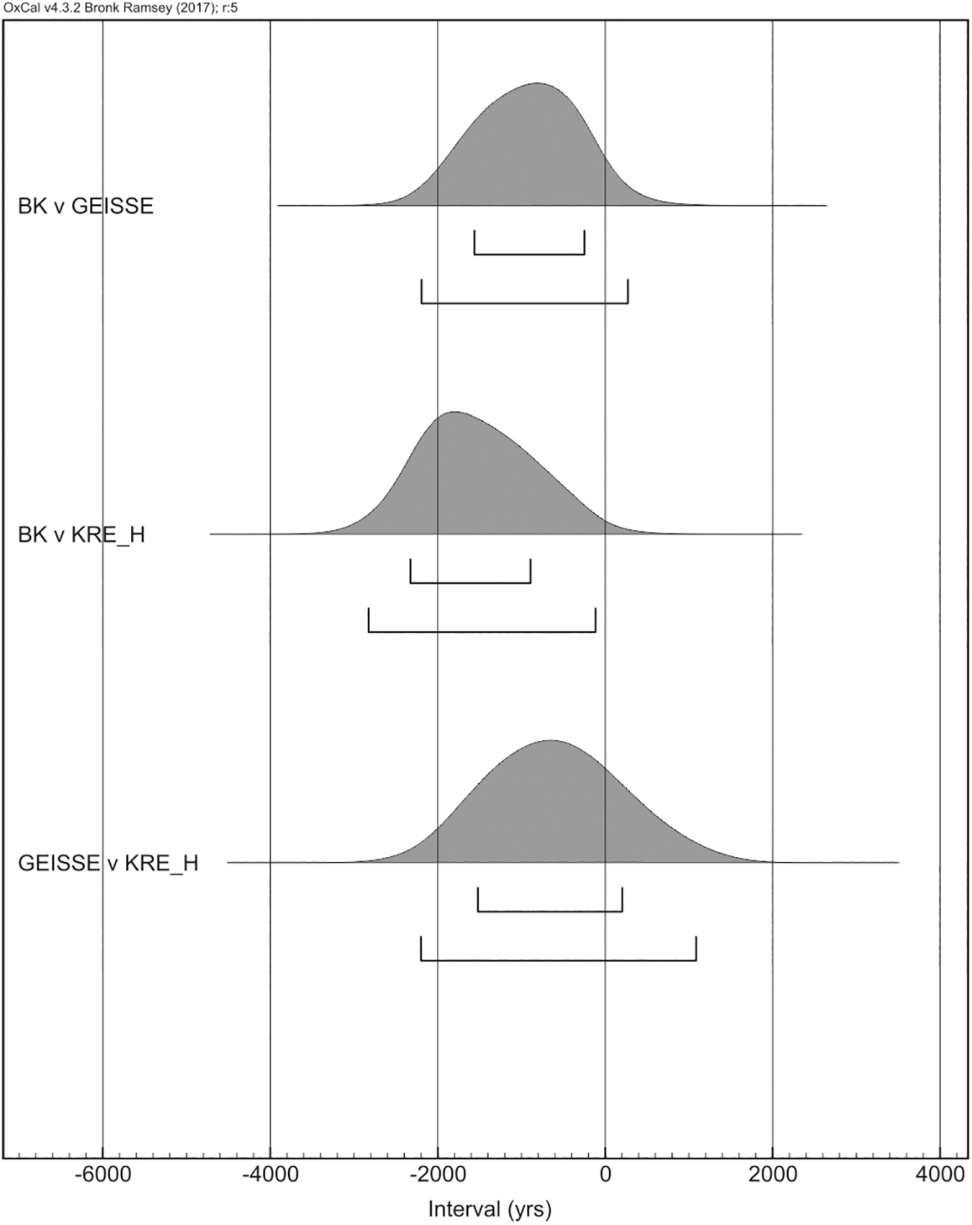
Results of the difference function for the three early sites with potential chronological overlap.

The linear regression calculated for the three models (Fig 2) show relatively high correlation values only for two sites: Geissenklosterle and Krems-Hundssteig, respectively with *r* = 0.66 (*p* < .05) and *r* = 0.57 (*p* < .05). The regression of Buran Kaya III as the origin point resulted in a very low correlation value (*r* = 0.36, *p* > .05). These results suggest that:

1. The Buran Kaya III date is likely erroneous and does not represent the beginning of the Gravettian in the region; this is suggested not only by the low correlation value and lack of statistical significance, but also because there is a huge geographical gap between Buran Kaya III and the next site, Krems-Hundssteig;
2. The origin of the Gravettian technology is more probable to have occurred in central Europe, as suggested by Otte and Keeley [7], likely in Germany in Geissenklosterle–this site has the earliest date in the region, the regression result is the highest we obtained with statistical significance, and the results from the Order (S2 Table) and Difference functions (Fig 3) also seem to indicate it as the earliest site;
3. The Geissenklosterle regression seems to indicate two fairly linear correlations between distance and age, parallel to each other, representing two slightly different speed of advances: one small group including mostly sites from SW France and Northern Spain representing a slower speed of advance; and a second, larger and faster group with the other sites;
4. Finally, the results seem to indicate that the propagation of the Gravettian techno-complex occurred in a fairly constant and slow spreading rate in most directions over the European landscape. This can be observed by the position of the sites in Fig 3, where the most distant sites appear at the same time distance from the probable origin. This is the case of Lapa do Picareiro and Vale Boi in Western Iberia and Mira in Eastern Europe, or Antolinako Koba in Northern Spain and Poiana Ciresului in Central-Eastern Europe.

The slope regression lines in the models from both sites, Geissenklosterle and Krems-Hundssteig, indicate a small difference in the speed ranges, respectively 0.66±0.18 km/yr and 1.02±0.38 km/yr (Table 2). When compared with other simulations [8] this low speed values likely represent the spread of the Gravettian cultural patterns through human demic dispersals.

**Table 2:** Correlation results for the Cost-distance models and respective speed of advance (in Kms).

Model	*r*	Slope	Standard Error Slope	Speed	Standard Error Speed	Speed of advance (in Km)
Geissenklosterle	0.657	1.5068	0.4191	0.6637	0.1816	0.48–0.85
Krems-Hundssteig	0.568	0.9834	0.3683	1.0168	0.3808	0.64–1.40

**Fig 4:**
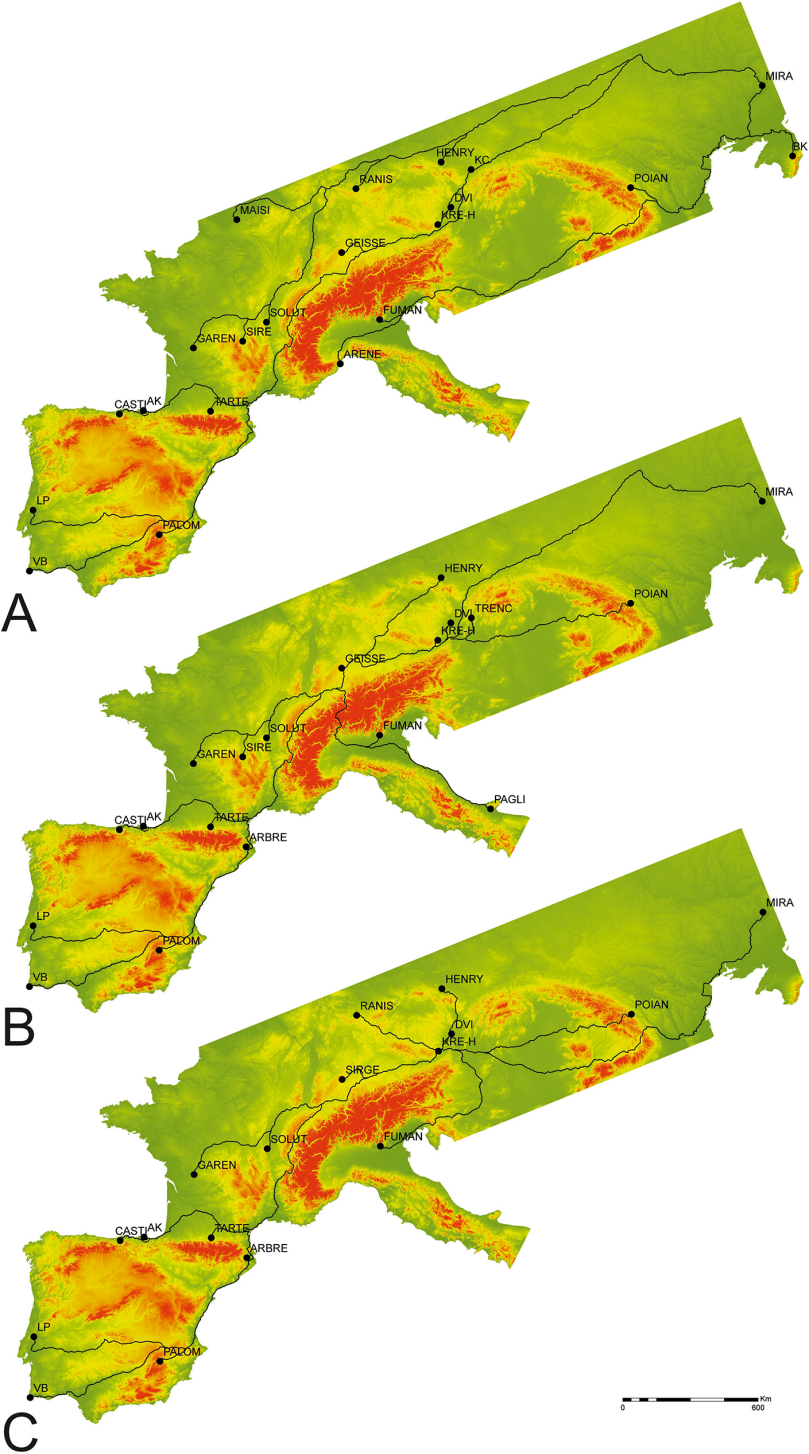
Map with site locations and optimal-path routes for the dispersal of the Gravettian techno-complex. A) Buran Kaya III Model; B) Geissenklosterle Model; C) Krems-Hundssteig Model.

Additionally, the high correlation demonstrated in the Cost-distance regression validates the high significance of the calculated Least-Cost Paths between sites as possible dispersion routes for Gravettian people. Fig 4B presents the modelling of all probable demic least-cost paths originating at Geissenklosterle and ending at each one of the remaining sites. The model is based on the idea that humans would have choose the best path (in this case the one requiring less physical effort avoiding as much as possible rugged paths) to reach an unknown destination [32].

The model helps to perceive that, regardless a similar rate of spread, while the plains were easily used as the main avenue for the dispersal in Central Europe, the entry into Iberia was likely through the edges of the Pyrenees (both on the North and Southern extremes), right against the Atlantic and Mediterranean shores avoiding, thus, the high ridged and rugged mountainous peaks, where the speed of advance was slower. It is likely thus that two different Iberian territories were occupied differently using different routes: the northern region with the Cantabrian Cordillera and its coastal lowlands, with a relatively difficult path; and the open access Eastern and Southern coastal landscapes of both Spain and Portugal. The model for Buran Kaya III, for example, represents a very different pattern (Fig 4A), where there are different parallel lines of optimal paths running to different parts of the European territory, as if there were various simultaneous waves-of-advance across the landscape.

## Discussion

Wave-of-advance studies for prehistoric phases, such as the Neolithization (e.g., [14, 15, 16]), the recolonization after major changing climatic events [12], or the new colonization of human-free regions (e.g., [13]), has changed through time, using approaches such as the Euclidean and Shortest-Path methods [14]. We used GIS to obtain faster and diverse data, that provided a very different view on the distances between sites and, more importantly, enables the development of hypothetical models for the possible rate of human dispersal across large surfaces such as the European territory. The same data facilitate the making of a map for each model (Fig 4) with possible early human migration and re-colonization routes, and paths which the Gravettian communities, corresponding to the first Upper Paleolithic techno-complex reaching all four corners of the European late Pleistocene wilderness, may have followed.

Except for the work of Otte and Keeley [7], where general directions were proposed for all main Upper Paleolithic techno-complexes, no other study has presented possible paths for the main migratory or colonization routes during the Upper Paleolithic. In face of the results here presented, with a very slow demic expansion of the Gravettian communities, Fig 4B represents a further step to the modeling process, showing that in the Iberia and Italian peninsulas, the penetration routes were likely coastal, avoiding the rugged mountain regions as well as occupying the areas with higher resource availability and diversity, and suffering less from climatic impacts. In the rest of the area, on the contrary, the tendency was likely that of cross-country the flat landscape of the central European plains, circumventing the main mountain systems.

The construction of the Cost-distance predictive models, grounded on optimal path theory, are justified by the assumptions described by Whitley and Burns for the early peopling of the Americas [33] where possible initial migration and dispersal paths are dependent on the basic resources for those hunter-gatherers: the herbivore prey species, in many cases migratory. The paths for South Carolina Paleoindian developed by Whitley and Burns [33], for example, are based on identical technological, economic and social circumstances than those of the Gravettian dispersals across Europe: the prehistoric hunter-gatherers likely used the paths following migratory and other herbivore herds, since human migration routes, and their settlement and subsistence patterns, developed initially depended on the access to and availability of hunting resources [32, 33]. According to Whitley and Burns, proximity is the key element to travel (in fact, to all spatially limited activities) while spatial knowledge is built on the frequency of local activities. In practical terms, travelling route decisions are based on landscape data acquisition by the hunter-gatherer direct visual contact of the immediate surroundings as well as on the visible landscape observed during both daily economic and scouting activities. Elements such as slope inclination and length, vegetation cover or physical barriers such as waterways, mountains or gorges, were important elements to consider for the dispersal paths of those hunter-gatherers [32, 33]. The direct result is an estimation of between 0.7 and 1 km annual speed rate across Europe, depending on the site origin, likely representing demic dispersal (for a detailed discussion see [8]). More importantly, the fact that the speed of advance to the Southwest is the same of the Northeast suggests that human settlement prior to the Gravettian expansion was identical on both directions, with likely similar conditions in prior human occupations, representing in any case either unpopulated or sparsely human populated regions. Thus, there was likely little or no competition for natural resources in those far regions for the geographical progression of AMH with Gravettian technology. One should note, however, that there are both geographical and chronological data gaps. These may be the result of lack of research or international publications as much as the reflex of true patterns. Different data may change radically the models presented here.

Nevertheless, the very slow rate of advance seen in our two models for the sites of Geissenklosterle and Krems-Hundssteig are on the lower limit of the predictions of the wave-of-advance model presented by Fort et al. [12] for prehistoric hunter-gatherers. The most plausible explanation for this is the presence of a very low demographic human density in Europe between 37 and 30 thousand years ago, perhaps lower than previous estimations for the Upper Paleolithic in Europe [34]. The differences in the correlation between the furthest away sites, both in the Northeast and Southwest, and the sites in central Europe and France, indicate that the rate of spread was faster in the distant regions. This scenario may confirm scenario #1 formulated by Wood et al. [6] in which Southern Iberia was depopulated at the time of AMH arrival there.

The use of GIS-based Cost-distance models increases the quality, diversity and accuracy of spatial limited activities, including the modelling of waves and rates of speed of advance, colonization and dispersal routes. Based on a combination of traditional wave-of-advance calculation methods with Least-Cost Path modelling, we presented here a further step in the application of spatial and demographic analyses to Upper Paleolithic data. The result was the construction of a pioneer wave-of-advance model for the first AMH group that reached all areas of the European ice-free territory, between 37 ka and 30ka years ago. The speed was likely around 0.7–1.0 km/yr and the slightly faster rate in both the extreme east and western regions, seem to suggest a colonization of landscapes with very low demography or even devoid of hominin competitors, AMH, Neanderthals or Denisovans in those regions. Hopefully, new sites and more absolute dates will confirm (or deny) this demographic model for the Gravettian and early AMH in Europe.

## Supporting information

S1 Table List of all Gravettian sites dated by AMS to more than 30 k calibrated years ago. Source: Radiocarbon Palaeolithic Europe Database v20.
(DOCX)

S2 Table Results of the Order command for all early Gravettian sites. (XLSX)
