# Supplementary material for: Early Upper Paleolithic colonization across Europe: Time and mode of the Gravettian diffusion
Source: PLoS One. 2017 May 24;12(5):e0178506. doi: 10.1371/journal.pone.0178506 (PMC5443572; doi:10.1371/journal.pone.0178506)
Supplement: S1 Table — Source: Radiocarbon Palaeolithic Europe Database v20. (DOCX) [file pone.0178506.s001.docx]

S1 Table. List of all Gravettian sites dated by AMS to more than 30 ka cal BP. Source: Radiocarbon Palaeolithic Europe Database v20 (*20*).

| **Site** | **Code** | **Layer** | **Longitude** | **Latitude** | **Country** | **Lab Code** | **Sample** | **Date** | **SD** | **Calibrated Interval (2 sigma)** | **Mean Calibrated BP** |
| --- | --- | --- | --- | --- | --- | --- | --- | --- | --- | --- | --- |
| Abri Pataud | PATAUD | 5 sublayer H3 back: superior | 1.0121 | 44.9379 | France | OxA-21586 | Bone | 28230 | 290 | 32966-31393 | 32180 |
| Antolinako Koba | AK | Lmbk sup | -2.6519 | 43.3710 | Spain | Beta-230279 | Bone | 27520 | 190 | 31643-31052 | 31348 |
| Arbreda | ARBRE | F | 2.7470 | 42.1611 | Spain | OxA-21782 | Bone | 28280 | 290 | 33030-31424 | 32227 |
| Brillenhohle | BRILL | VII | 9.7782 | 48.4060 | Germany | KIA-19549 | Bone | 27030 | 180 | 31300-30825 | 31063 |
| Buran Kaya III | BK | 6-2 and 6-1 | 34.3833 | 45.0170 | Ukraine | GrA-40485 | Bone | 34050 | 260 | 39195-37860 | 38528 |
| Combe Sauniere | COMBE | VI | 0.8836 | 45.2307 | France | OxA-6514 | - | 27880 | 440 | 32971-31060 | 32016 |
| Dolni Vestonice II-05 | DVI5 | hearth (5) | 16.6361 | 48.8828 | Czech Republic | OxA-17813 | Charcoal | 27080 | 140 | 31294-30880 | 31087 |
| Dolni Vestonice IIa | DVI | 4 | 16.6361 | 48.8828 | Czech Republic | OxA-27333 | Charcoal | 31650 | 280 | 36159-34940 | 35550 |
| El Castillo | CASTI | 14 | -3.9650 | 43.2900 | Spain | Beta-298432 | Bone | 29740 | 190 | 34214-33559 | 33887 |
| Fumane | FUMAN | D1d | 10.9666 | 45.5062 | Italy | OxA-17571 | Charcoal | 31590 | 160 | 35916-35041 | 35479 |
| Geissenklosterle | GEISSE | I c | 9.7804 | 48.3934 | Germany | OxA-18718 | Bone | 33380 | 390 | 38602-36536 | 37569 |
| Grotta Arene Candide | ARENE | P12, hearth VI of Cardini | 8.3330 | 44.1667 | Itally | LTL3769A | Charcoal | 27381 | 200 | 31537-30989 | 31263 |
| Henrykow 15 | HENRY | 9 | 16.9960 | 50.6432 | Poland | Poz-60000 | Charcoal | 31550 | 350 | 36181-34772 | 35477 |
| Hohle Fels, Hohler Fels | HOHLE | II C- 11 | 9.7541 | 48.3792 | Germany | OxA-4599 | Bone | 28920 | 440 | 33912-31781 | 32847 |
| Huccorgne - Hermitage | HUCCOR | 4 | 5.1806 | 50.5625 | Belgium | CAMS-5891 | Bone | 28390 | 430 | 33464-31395 | 32430 |
| Komarowa Cave | KC | C | 18.5117 | 49.9773 | Poland | GdA-94 | Bone | 28500 | 500 | 33646-31406 | 32526 |
| Krems-Hundssteig | KRE-H | AH 3 | 15.6016 | 48.4148 | Austria | VERA-2289 | Charcoal | 32810 | 450 | 38315-35933 | 37124 |
| Krems-Wachtberg | KRE-W | AH4 | 15.5993 | 48.4149 | Austria | VERA--3939 | Charcoal | 28750 | 270 | 33592-31935 | 32764 |
| Lapa do Picareiro | LP | Z | -8.6520 | 39.5305 | Portugal | Wk-32280 | Bone | 29054 | 224 | 33769-32690 | 33230 |
| Le Sire | SIRE | - | 3.2333 | 45.7000 | France | Beta-145820 | Bone | 29350 | 310 | 34095-32834 | 33465 |
| Les Garennes | GAREN | - | 0.4200 | 45.6811 | France | Beta-216143 | Bone | 28410 | 230 | 33061-31587 | 32324 |
| Maisieres Canal | MAISI | unity M H (archaeological layer) | 3.9803 | 50.4804 | Belgium | OxA-17962 | Bone | 29060 | 170 | 33696-32826 | 33261 |
| Mira | MIRA | Lower, II/2 | 35.1024 | 47.6750 | Ukraine | CURL-15795 | Charcoal | 27400 | 260 | 31706-30924 | 31315 |
| Paglicci | PAGLI | 23 a | 15.6152 | 41.6540 | Italy | UtC-1414 | - | 28100 | 400 | 33103-31210 | 32157 |
| Poiana Ciresului | POIAN | IV | 26.3277 | 46.9306 | Romania | Erl-11859 | Charcoal | 27321 | 234 | 31551-30921 | 31236 |
| Palomar | PALOM | II (3.75-4.15 m) | -2.2459 | 38.3975 | Spain | Beta-185412 | - | 28050 | 230 | 32633-31332 | 31983 |
| Ranis | RANIS | VI | 11.5632 | 50.6613 | Germany | OxA-13046 | Bone | 31780 | 330 | 36344-34965 | 35655 |
| Sirgenstein | SIRG | II | 9.7617 | 48.3853 | Germany | KIA-13079 | Bone | 27250 | 180 | 31426-30941 | 31184 |
| Solutre | SOLUT | en magma | 4.7260 | 46.2976 | France | SR-5595/CAMS-70703 | - | 28420 | 160 | 32918-31719 | 32319 |
| Tarte | TARTE | 1b-c | 0.9828 | 43.1072 | France | Ly-2105-OxA | Bone | 28410 | 150 | 32887-31729 | 32308 |
| Trencianske Bohuslavice-Pod Tureckom | TRENC | IV? | 17.8536 | 48.8001 | Slovakia | GrA-6139 | Charcoal | 29910 | 260 | 34510-33605 | 34058 |
| Vale Boi | VB | 6 terrace | -8.8150 | 37.0944 | Portugal | Wk-32146 | Shell | 28321 | 422 | 33389-31354 | 32372 |
| Willendorf II | WILEND | 6 / B4 | 48.3230 | 15.3990 | Germany | GrA-895 | - | 27620 | 230 | 31929-31053 | 31491 |
